# Application of Artificial Neural Network and Support Vector Machines in Predicting Metabolizable Energy in Compound Feeds for Pigs

**DOI:** 10.3389/fnut.2017.00027

**Published:** 2017-06-30

**Authors:** Hamed Ahmadi, Markus Rodehutscord

**Affiliations:** ^1^Bioscience and Agriculture Modeling Research Unit, College of Agriculture, Tarbiat Modares University, Tehran, Iran; ^2^Institut für Tierernährung, Universität Hohenheim, Stuttgart, Germany

**Keywords:** pig, compound feed, metabolizable energy, artificial neural network, support vector machines

## Abstract

**Background:**

In the nutrition literature, there are several reports on the use of artificial neural network (ANN) and multiple linear regression (MLR) approaches for predicting feed composition and nutritive value, while the use of support vector machines (SVM) method as a new alternative approach to MLR and ANN models is still not fully investigated.

**Methods:**

The MLR, ANN, and SVM models were developed to predict metabolizable energy (ME) content of compound feeds for pigs based on the German energy evaluation system from analyzed contents of crude protein (CP), ether extract (EE), crude fiber (CF), and starch. A total of 290 datasets from standardized digestibility studies with compound feeds was provided from several institutions and published papers, and ME was calculated thereon. Accuracy and precision of developed models were evaluated, given their produced prediction values.

**Results:**

The results revealed that the developed ANN [*R*^2^ = 0.95; root mean square error (RMSE) = 0.19 MJ/kg of dry matter] and SVM (*R*^2^ = 0.95; RMSE = 0.21 MJ/kg of dry matter) models produced better prediction values in estimating ME in compound feed than those produced by conventional MLR (*R*^2^ = 0.89; RMSE = 0.27 MJ/kg of dry matter).

**Conclusion:**

The developed ANN and SVM models produced better prediction values in estimating ME in compound feed than those produced by conventional MLR; however, there were not obvious differences between performance of ANN and SVM models. Thus, SVM model may also be considered as a promising tool for modeling the relationship between chemical composition and ME of compound feeds for pigs. To provide the readers and nutritionist with the easy and rapid tool, an Excel^®^ calculator, namely, SVM_ME_pig, was created to predict the metabolizable energy values in compound feeds for pigs using developed support vector machine model.

## Introduction

In the field of pig nutrition, several systems for energy evaluation of feedstuff are in use based on digestible, metabolizable, or net energy. The measurements of gaseous energy losses and heat expenditure are expensive and may not be made routinely, thus, many evaluation systems try to calculate the energy value of a specific feed using the digestibility of its nutrients (1). However, because the ingredient composition of the compound feeds and their digestibility are usually unknown, the common energy evaluation of compound feeds cannot be done this way. Thus, several models were developed that predict the energy value from the analyzed chemical composition of the feed. Models for predicting the energy value of a pig diet based on analyzed crude nutrients have been previously published (2–6). All of the published models were developed based on compound feed data using a conventional approach of multiple linear regression (MLR) to describe the relationship between chemical composition of the feed and its metabolizable energy (ME) content. These models have different numbers of input variables, which produce a wide range of accuracy and precision. For animal nutritionist working in the field of feed evaluation, the search to find and apply some alternative and more efficient approaches for data modeling have always been an interesting subject. Approaches of modeling based on artificial intelligence, such as artificial neural networks (ANNs) and support vector machines (SVM), may be used to process feed data. They possess the ability of developing relationships between input(s) and output(s) variables using a set of data samples and do not require *a priori* knowledge of the governing processes (7, 8). The ANN models have been used in several feedstuff evaluation approaches in the field of animal nutrition (9–11); however, the use of SVM technology in animal and feed research has not been explored as yet. The SVM is a method proposed by Vapnik (12) and is processed based on statistical learning theory which seems a promising approach for modeling multivariate data. The SVM is actually a learning system that uses a hypothesis space of linear functions in a high-dimensional feature space, trained with a learning algorithm from the optimization theory (7). Unlike the ANN that try to minimizing the error on the training data, the SVM attempts to minimize the upper bound on the generalization error based on the principle of structural risk minimization, which has been found, in several cases, to be superior to the empirical risk minimization principle employed in ANN (7, 13).

Our objectives were to (1) develop the MLR, ANN, and SVM models to predict ME in compound feeds for pigs based on an established energy evaluation system from analyzed contents of crude protein (CP), ether extract (EE), crude fiber (CF), and starch based on an established energy evaluation system; (2) compare the performance of developed models in term of accuracy and precision given their produced prediction values; (3) apply the developed models to rank the relative importance of dietary nutrients on ME content; and (4) create an Excel^®^ ME calculator, which can be used by the nutritionists to predict ME of compound feed samples.

## Materials and Methods

This study was based on data obtained from different laboratories and institutes and did not involve the use of animals; thus, Institutional Animal Care and Use Committee approval was not obtained.

### Data Set

A detailed description of the animals, diets, and measurements made was given by Bulang and Rodehutscord (6). Briefly, research institutions in Germany contributed their data from digestibility trials using compound feeds for pigs. The trials had been conducted in accordance with the guidelines for pig studies on the energy value of feeds (14), with the exception that no restriction in body weight had been implemented. Each dataset contained information about the ingredient composition of the compound feed, its dry matter concentration, and the concentrations of organic matter, CP, EE, CF, N-free extract, starch, and sugar (grams per kilogram of dry matter in all cases). Finally, a total of 290 datasets from digestibility studies with these compound feeds were provided. The concentration of ME in all compound feeds was calculated for each feed from digestible nutrients using Eq. 3 of Ref. (1). It was calculated and presented as megajoules per kilogram of dry matter. The equation is as follows:

$$\begin{array}{c} {\text{ME}}_{\text{s}}=0.0205\times \text{DCP}(\text{g})+0.0398\times \text{DCF}(\text{g})+0.0173\times \text{St}(\text{g})+0.0160\times \text{Su}(\text{g})+0.0147\times \\ (\text{DOM}-\text{DCP}-\text{DCF}-\text{St}-\text{Su})(\text{g}), \end{array}$$

where OM, organic matter; DCP, digestible crude protein; DCF, digestible crude fat; St, starch; Su, sugar. The unit of regression coefficient is megajoules per gram.

A large proportion of the compound feeds consisted of wheat grain (56%), barley grain (19%), and a mixture of both (15%) as main ingredients [Table 1 of Ref. (6)]. The collected information was organized in an Excel spreadsheet for further processing. To keep the model simple, among all information, the CP, EE, CF, and starch were used as input variables while ME was considered as model output.

**Table 1:** Information and descriptive statistics for the data used in modeling process.

	Train set (*n* = 202)				Validation set (*n* = 88)				All data (*n* = 290)			
	Min	Max	Mean	SD	Min	Max	Mean	SD	Min	Max	Mean	SD
**Model inputs**												
CP (g/kg)	114.0	245.0	199.5	24.8	122.0	243.5	196.8	24.2	114.0	245.0	198.7	24.6
EE (g/kg)	12.0	98.0	35.5	12.4	12.0	107.0	33.5	13.1	12.0	107.0	34.9	12.6
CF (g/kg)	27.0	139.5	44.1	16.9	23.0	130.5	44.7	19.6	23.0	139.5	44.2	17.7
Starch (g/kg)	151.1	618.0	453.9	71.2	95.4	658.0	461.3	94.7	95.4	658.0	456.1	78.9
**Model output**												
ME (MJ/kg)	10.4	16.6	15.1	0.8	11.0	16.0	15.0	0.9	10.4	16.6	15.0	0.8

*CP, crude protein; EE, ether extract; CF, crude fiber; ME, metabolizable energy for pigs*.

*Values for all criteria presented as on dry matter basis. Dataset originally published in Ref. (6)*.

### Model Development

The original dataset corresponding to 290 feeding trials using compound feed was randomly divided into two sets of training and validation with 202 (70%) and 88 (30%) samples, respectively. The training set was used to build MLR, ANN, and SVR models while the validation set was used as unforeseen data to check the generalization of developed models. Description of data used for the modeling and evaluating processes were summarized in Table 1.

### Multiple Linear Regression

A MLR model was defined as the following general equation,

(1) $${\overset{\wedge }{y}}_{i}=\beta_{0}+\sum_{i=1}^{n}\beta_{i}X_{i}+e_{i},\;i=1,2,\ldots n$$

where: ${\overset{\wedge }{y}}_{i}$ is the ME in the *i*^th^ sample, *X_i_* is the value corresponding to input variables (CP, EE, CF, and starch concentration in compound feed) in the *i*^th^ sample (assumed to be a known constant measured without error), β*_0_* is the overall intercept, β*_i_* is the linear coefficient for input variables, and *e_i_* is the residual error assumed to be normal [*N* ~ (0, σ2)].

Data related to the training set were fitted to the Eq. 1 by means of the REG procedure of Ref. (15). Using analysis of variance and corresponding absolute *t* value (|*t* value|) of the model coefficients, a process of the sensitivity analysis has been performed on the developed ME models to evaluate which chemical component is considered more important during the modeling process. A more important model term has a higher |*t* value|. Thus, the input variables may be ranked in the order of importance (16).

### ANN Model

An algorithm of the feed forward three-layer back propagation network was chosen and considered in constructing the ANN model (17). Hyperbolic tangent sigmoid (*tansig*) and linear (*purelin*) functions were used as the transfer function for the hidden and output layers, respectively. A Levenberg–Marquardt algorithm for back propagation with a gradient descent and momentum weight and bias learning function, was used to train the network (17). The mean squared error with level of 0.005 was used as the performance function, and training was terminated after 800 epochs or iterations of the network. Four input variables corresponding to CP, EE, CF, and starch were used as units in the input layer of the ANN model. The 202 data samples (training set) were used to train the network. Prior to training, the data set (input and output data) was normalized within the range [–1, 1]. This is to simplify the problem for the network, to achieve fast convergence minimum mean squared error, and to ensure the fall of targets (output data) into the specific range that the new feed-forward network can be reproduced (17, 18).

The obtained ANN model was utilized in the further process of sensitivity analysis to find which input variable is considered more important by the model. The sensitivity of ME against the dietary chemical fractions was determined using the following criteria (19, 20): the variable sensitivity error (VSE) value indicates the performance [in term of root mean square error (RMSE)] of the developed ANN model if that variable is unavailable. The value of variable sensitivity ratio (VSR) is a relative indication of the ratio between the VSE and the RMSE value of ANN model when all variables are available. A more important variable has a higher VSR value. Thus, based on the obtained VSR values, the input variables may be ranked in the order of importance.

Commercially available software, Matlab^®^ R2016a (21), was used to write the mathematical code for developing and evaluating the ANN model. The developed program is actually a modified source code of an ANN algorithm which was previously applied by Ahmadi and Golian (16).

### Support Vector Regression

Least squares support vector machine (LS-SVM) is an optimized algorithm based on the standard SVM reported by Suykens et al. (22). The LS-SVM has the ability for linear and non-linear multivariate calibration and solves the multivariate problems in a fast and efficient way (13). In this approach, a linear set of equations instead of a quadratic programming problem is used to obtain the support vectors (23). The LS-SVM adopts a least squares linear system as the loss function and is applied in the pattern recognition and non-linear evaluation. It is capable of learning in a high-dimensional feature space and needs fewer training data than those needed to train an ANN model. In the LS-SVM algorithm, a non-linear mapping function φ (·) is applied for constructing the regression model, whereas the input data are mapped to a higher dimensional feature space. When the LS-SVM is used as a soft testing tool, a new optimization problem is formulated in the case of structural risk minimization. The Lagrange function is then adopted to solve this optimization problem. In the SVM or LS-SVM process, the proper kernel function and the best kernel parameters need to be determined. However, no systematic methodology is available for prior selection of the kernel function. In our work, the most common kernel function, namely, radial basis function (RBF), was selected (7, 22). The RBF kernel can reduce the computational complexity of the training procedure while giving good performance under general smoothness assumptions (22). A grid-search technique was applied to find the optimal parameter values; these included the regularization parameter gam (γ) and the RBF kernel function parameter sig^2^ (σ^2^), which is the bandwidth in the common case of the RBF kernel. The parameter γ determines the trade-off between *structural risk minimization* and *empirical risk minimization* and is important to improve the generalization performance of the model (7). The parameter σ^2^ controls the value of the function error. A small value of σ^2^ can lead to overfitting, whereas a large value of σ^2^ will make the model simpler but not accurate. Moreover, σ^2^ reflects the sensitivity of the LS-SVM model to noise from the input variables (24). In our study, these parameters were optimized with values of γ in the range of 0.1–20 and with values of σ^2^ in the range of 1–100, with adequate increments by the grid-search technique of leave-one out cross-validation. After the LS-SVM model with high prediction accuracy and stability was built, the same process of sensitivity analysis as done in ANN modeling was done to rank the input variables according to their importance.

Mathematical code for formulating and evaluating the LS-SVM model was developed using Matlab^®^ R2016a (21). The LS-SVM toolbox ver. 1.8 (25) was applied with developed functions to derive all calculations.

### Model Evaluation and Comparison

Performance of the models was evaluated using criteria, which are commonly used to evaluate forecasting models (26). The *R*^2^ computed as,

$$R^{2}=1-\frac{\sum_{i=1}^{n}{\left(y_{i}-{\overset{\wedge }{y}}_{i}\right)}^{2}}{\sum_{i=1}^{n}{\left(y_{i}-{\bar{y}}_{i}\right)}^{2}}$$

Root mean squared error computed as,

$$\text{RMSE}=\sqrt{\frac{\sum_{i=1}^{n}{\left({\overset{\wedge }{y}}_{i}-y_{i}\right)}^{2}}{n}}$$

Mean absolute percentage error (MAPE) computed as,

$$\text{MAPE}=\frac{\sum_{i=1}^{n}\left|\frac{\left(y_{i}-{\overset{\wedge }{y}}_{i}\right)}{y_{i}}\right|}{n}\times 100$$

and the bias computed as,

$$\text{Bias}=\frac{\sum_{i=1}^{n}\left[y_{i}-{\overset{\wedge }{y}}_{i}\right]}{n}$$

where *n* is the number of observations, and *y_i_*, ${\overset{\wedge }{y}}_{i}$, and ${\bar{y}}_{i}$ are equal to observed, predicted, and average observed values of ME. Model adequacy was also assessed using plots of residuals (observed minus predicted) against predicted values of *y* to test for linear prediction bias (27).

## Results

**Figure 1:**
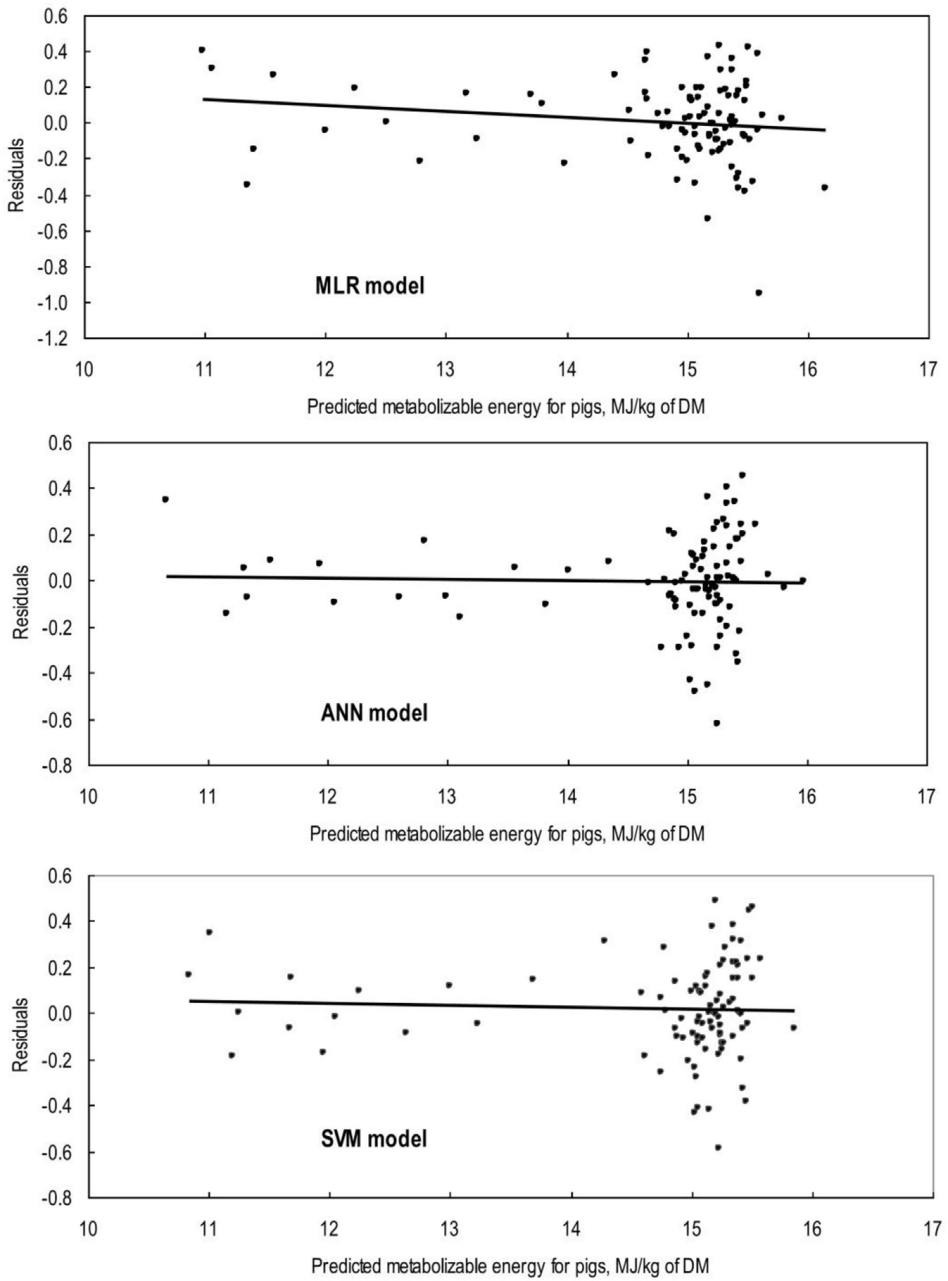
Plot of residuals (validation set; *n* = 88) against predicted values of metabolizable energy for pigs (ME) from the multiple linear regression (MLR), artificial neural network (ANN), and support vector machines (SVM) models. The line represents the regression of residuals on MLR predicted ME (*Y* = 0.477 − 0.032 × predicted ME; *R*^2^ = 0.01; *P* = 0.26), on ANN predicted ME (*Y* = 0.067 − 0.005 × predicted ME; *R*^2^ = 0.00; *P* = 0.85), and on SVM predicted ME (*Y* = 0.146 − 0.009 × predicted ME; *R*^2^ = 0.00; *P* = 0.73).

**Figure 2:**
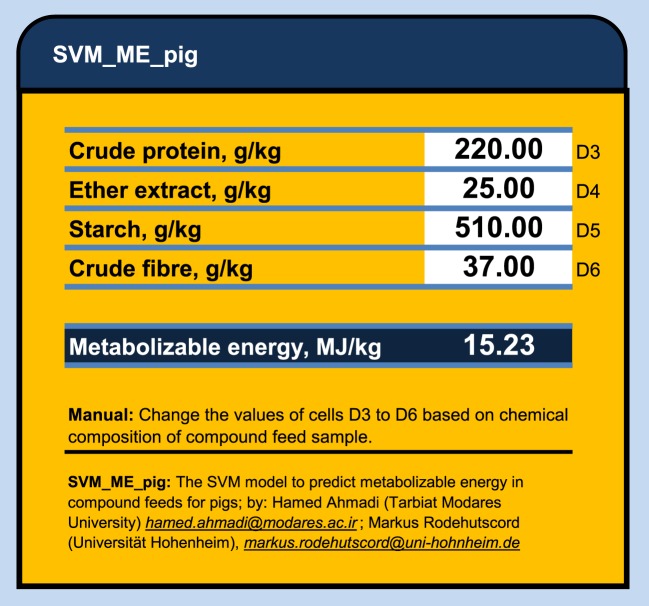
The SVM_ME_pig: an Excel^®^ calculator to predict the metabolizable energy values in compound feeds for pigs using support vector machine model.

The first task in regression analysis was to estimate the coefficients of Eq. 1 by least squares method using the training data set and to obtain information about the fit in the form of an analysis of variance. The parameter estimates with *P* and *t* values were shown in Table 2. All parameter estimates were highly different from 0 (*P* < 0.001). The fit of the MLR model was also expressed by the *R*^2^ value which was found to be 0.89 and 0.92 for training and validation sets (Table 3), respectively, indicating that 92% of the variability in the responses could be explained by the developed regression model when it is faced with unforeseen data. The plot of residuals (observed minus predicted) in validation dataset against predicted values showed no evidence of any linear or non-linear prediction bias for the model (Figure 1). The calculated absolute *t* value (|*t* value|) may indicate to what extent each model term was contributing to the statistical fit so that the greater the magnitude of |*t* value|, the more significant was the corresponding coefficient. The coefficient estimates for ME model and the corresponding absolute *t* values (Table 2) suggest that among the investigated inputs, CF content of feed had the largest effect on ME (|*t* value| = 14.9), followed by EE (|*t* value| = 9.9), starch (|*t* value| = 6.6), and CP (|*t* value| = 6.2). The goodness of fit statistical values derived from the ANN and SVM models to predict the ME are shown in Table 3. The plot of residuals (in validation dataset) against predicted values showed no evidence of any linear or non-linear prediction bias for the models (Figure 1), implying a good agreement between the observed and predicted values of ME for the dataset which is fed to the developed ANN and SVM models. Through sensitivity analysis of ANN and SVM models, the relative importance of input variables was determined using the entire 290 lines of data (training and validation) to calculate the overall VSE and VSR. The VSR obtained for the model output (ME), with respect to dietary levels of CP, EE, CF, and starch are shown in Table 4. Analysis of the ANN model indicated that the ME was more sensitive to CF concentration (VSR = 26.9), followed by starch (VSR = 7.7), CP (VSR = 2.8), and EE (VSR = 2.7), while in the SVM model the ranking of input variables according to their importance were as CF (VSR = 18.6), starch (VSR = 7.1), EE (VSR = 3.2), and CP (VSR = 2.9). The CF content of feed was obviously the most important predictor in these two models as well as it was reported in several past studies. Finally, using the selected developed SVM model, an Excel^®^ ME calculator, namely, SVM_ME_pig, was created (Figure 2). It is provided in Supplementary Material.

**Table 2:** Coefficient estimates for the multiple linear regression model fitted to data on metabolizable energy (MJ/kg of dry matter) for pigs.

	Coefficient	SE	*t* value	*P*-value
Intercept	13.126	0.4694	28.0	***
Crude protein (g/kg)	0.007	0.0011	6.2	***
Ether extract (g/kg)	0.016	0.0016	9.9	***
Crude fiber (g/kg)	−0.031	0.0021	−14.9	***
Starch (g/kg)	0.003	0.0005	6.6	***

***P < 0.001.

**Table 3:** Computed goodness-of-fit values on multiple linear regression (MLR), artificial neural network (ANN), and support vector machines (SVM) models of metabolizable energy for pigs.

	Train set (*n* = 202)			Validation set (*n* = 88)		
	MLR	ANN	SVM	MLR	ANN	SVM
Goodness-of-fit criteria						
*R*^2^	0.89	0.95	0.95	0.91	0.96	0.94
RMSE (MJ/kg of dry matter)	0.27	0.19	0.21	0.23	0.20	0.21
MAPE (%)	1.31	1.00	1.20	1.19	1.01	1.06
Bias (MJ/kg of dry matter)	−0.0006	0.0001	0.0050	−0.0031	−0.0080	0.0133

*RMSE, root mean square error; MAPE, mean absolute percentage error*.

**Table 4:** The overall (training and testing sets; *n* = 290) sensitivity analysis of input variables in artificial neural network (ANN), and support vector machines (SVM) models of metabolizable energy for pigs.

		Input variables			
Model		Crude protein	Ether extract	Crude fiber	Starch
ANN	Variable sensitivity ratio (VSR)	2.8	2.7	26.9	7.7
	Rank	3	4	1	2
SVM	VSR	2.9	3.2	18.6	7.1
	Rank	4	3	1	2

## Discussion

In many applications, the final goal of ANN and SVM processing is to find models that give more precise and accurate prediction value(s) of the output variable(s). The comparison of actual and predicted output values may describe the behavior of the ANN and SVM models based on the investigated inputs. Both ANN and SVM model could accurately (*R*^2^ = 0.96 and 0.94, respectively) predict the ME in the validation data set that were not used during the training processes. Moreover, the trained models of ME had relatively balanced statistical values for the two subsets of training and validation (Table 3), suggesting that the overlearning had not occurred during the training process and that the developed models had good generalizability when they faced a totally unseen data set (19). To evaluate the performance of developed models, the *R*^2^ value is used as a common criterion to judge about the “accuracy” of a specific model based on its prediction, while the RMSE and MAPE are commonly used to show the “precision” of a model based on residual analysis. Therefore, it is preferred to use a combination of criteria to conclude and/or compare overall performance of models. In modeling ME based on concentrations of feed chemical component, the goodness of fit in terms of *R*^2^, RMSE, and MAPE corresponding to MLR, ANN, and SVM models showed a relatively higher accuracy and precision of prediction for ANN and SVM model as compared with MLR model for both training and validation data sets (Table 3). The bias value implies the magnitude of the model over/under estimation regarding to the average of observed values. Some very small bias values (Table 3) may be observed in predicted values by all three models. However, these bias were not statistically significant as they could be seen in Figure 1 and corresponding calculated regression line on predicted values vs. residuals in the validation datasets. The better performance of ANN and SVM models over MLR model is mainly because the conventional MLR requires the specification of a linear function to be regressed, thus, the flexibility of regression equation may be extremely limited (28). Alternatively, the different kind of ANN and SVM models are good at fitting functions and recognizing patterns in different kind of data. In fact, there is a proof that a fairly simple ANN or SVM may fit many practical function (17). It is believed that the ANN and SVM method would require much more number of data samples than MLR to build an efficient model. However, ANN and SVM models may also work well when enough dataset is available and the data are statistically well distributed in the input domain.

The main advantage of ANN and SVM compared to conventional regression are: (1) The ANN and SVM models do not require a prior specification of suitable fitting function and (2) ANN and SVM have a universal approximation capability and it can approximate almost all kinds of non-linear functions including quadratic functions, whereas MLR is useful only for linear approximations (18, 29). However, there are some limitations for both the ANN and SVM modeling techniques. In these techniques, standardized coefficients corresponding to each variable may not be easily calculated and presented as they are in regression models. The ANN and SVM analyses produce matrix of weights, which are difficult to interpret as they usually are affected by the program used to generate them. Thus, they actually use a “black box” approach, which does not offer complete insight into the internal workings of the model or information for evaluating the interaction of inputs (8). In addition, there are some difficulties in sharing the developed ANN and SVM models with other researchers. In MLR model, one needs only to know the coefficients of the generated model and to perform simple calculations to predict an output (e. g., ME in our case). But, to share developed ANN and SVM models, one needs to provide either a copy of the trained model or the connection weight matrices, which might be extremely large and complex, while to run ANN or SVM models one also needs some especial program or software. In this study, we export the developed SVM models as a C++ code and SVM_ME_pig Excel^®^ ME calculator to share them with the readers who might be interested to duplicate the results or to predict a new output (ME for pig) based on dietary chemical components. This spreadsheet is accessible via Supplementary Material. The SVM_ME_pig (Figure 2) provides the nutritionist with an efficient and user-friendly tool to predict the metabolizable energy values in compound feeds for pigs using support vector machine model. The only required information to obtain a given ME (megajoules per kilogram of dry matter) value is the chemical contents of CP, EE, CF, and starch (grams/kilogram of dry matter) in given feed sample.

## Conclusion

The present study proposes the ANN and SVM approaches to predict ME of compound feeds for pigs given levels of feed chemical compositions of CP, EE, CF, and starch. Developed ANN and SVM models produce relatively better prediction values in estimating ME in compound feed than those produce by conventional MLR, while there are no obvious differences between performance of ANN and SVM models. The results suggest that SVM methods may be able to enhance our ability to accurately predict energy contents of diets in order to achieve optimal situation in pig nutrition. The developed and presented Excel^®^ metabolizable calculator provides the nutritionist with an efficient and user-friendly tool to predict the metabolizable energy values in compound feeds for pigs using support vector machine model.

## Author Contributions

HA conceived the study, developed models, analyzed data, and drafted the manuscript. MR designed the research, provided and interpreted data set, and revised manuscript. All authors reviewed the manuscript for intellectual content and gave final approval for the version to be published.

## Conflict of Interest Statement

The authors declare that the research was conducted in the absence of any commercial or financial relationships that could be construed as a potential conflict of interest.

## Abbreviations

ANN, artificial neural network; CF, crude fiber; CP, crude protein; EE, ether extract; LS-SVM, least squares support vector machine (LS-SVM); MAPE, mean absolute percentage error; ME, metabolizable energy; MLR, multiple linear regression; RBF, radial basis function; RMSE, root mean square error; SVM, support vector machines; VSE, variable sensitivity error; VSR, variable sensitivity ratio.
